# Tracking embryonic hematopoietic stem cells to the bone marrow: nanoparticle options to evaluate transplantation efficiency

**DOI:** 10.1186/s13287-018-0944-8

**Published:** 2018-07-27

**Authors:** Sean K. Sweeney, Gohar S. Manzar, Nicholas Zavazava, Jose G. Assouline

**Affiliations:** 1 Department of Biomedical Engineering, University of Iowa, 5601 Seamans Center for the Engineering Arts and Sciences, Iowa City, IA 52242 USA; 2 NanoMedTrix, LLC, University of Iowa BioVentures Center, 2500 Crosspark Road, Coralville, IA 52241 USA; 3 Mayo Clinic College of Medicine, 200 First St. SW, Rochester, MN 55905 USA; 4 Department of Internal Medicine, University of Iowa, 200 Hawkins Drive, Iowa City, IA 52242 USA; 5 Iowa City VA Health Care System, 601 Highway 6 W, Iowa City, IA 52246 USA

**Keywords:** Murine embryonic stem cells, Hematopoietic progenitor cells, Bone marrow transplant, Mesoporous silica nanoparticles, Magnetic resonance imaging

## Abstract

**Background:**

As the prevalence of therapeutic approaches involving transplanted cells increases, so does the need to noninvasively track the cells to determine their homing patterns. Of particular interest is the fate of transplanted embryonic stem cell-derived hematopoietic progenitor cells (HPCs) used to restore the bone marrow pool following sublethal myeloablative irradiation. The early homing patterns of cell engraftment are not well understood at this time. Until now, longitudinal studies were hindered by the necessity to sacrifice several mice at various time points of study, with samples of the population of lymphoid compartments subsequently analyzed by flow cytometry or fluorescence microscopy. Thus, long-term study and serial analysis of the transplanted cells within the same animal was cumbersome, making difficult an accurate documentation of engraftment, functionality, and cell reconstitution patterns.

**Methods:**

Here, we devised a noninvasive, nontoxic modality for tracking early HPC homing patterns in the same mice longitudinally over a period of 9 days using mesoporous silica nanoparticles (MSNs) and magnetic resonance imaging.

**Results:**

This approach of potential translational importance helps to demonstrate efficient uptake of MSNs by the HPCs as well as retention of MSN labeling in vivo as the cells were traced through various organs, such as the spleen, bone marrow, and kidney. Altogether, early detection of the whereabouts and engraftment of transplanted stem cells may be important to the overall outcome. To accomplish this, there is a need for the development of new noninvasive tools.

**Conclusions:**

Our data suggest that multifunctional MSNs can label viably blood-borne HPCs and may help document the distribution and homing in the host followed by successful reconstitution.

**Electronic supplementary material:**

The online version of this article (10.1186/s13287-018-0944-8) contains supplementary material, which is available to authorized users.

## Background

Embryonic stem (ES) cells have the potential to revolutionize regenerative medicine due to their ability to derive specialized cell types of all three germ layers [1]. A major application for ES cells has been the development of transplantable hematopoietic progenitor cells (HPCs) for the treatment of leukemia and other hematological malignancies [2]. Currently, clinical treatment may involve transplant of allogeneic hematopoietic stem cells (HSCs). However, the possible occurrence of severe complications such as graft versus host disease (GvHD), the requirement for immunosuppression and harsh preconditioning regimens, as well as the difficulty of organizing appropriate histocompatible matches between donors and recipients all limit the wide application of these therapies [2]. In contrast, ES cells can give rise to multipotent HPCs that can generate all hematopoietic lineages upon engraftment while being educated in the host’s own thymus, effectively eliminating the risk for GvHD. Additionally, these cells are poorly immunogenic and antigenic, giving them an “immuno-privileged” status that precludes immunosuppressive measures normally requisite for regular HSC transplant procedures [3]. We recently reported the establishment of robust multilineage chimerism in mice across major histocompatibility complex (MHC) barriers, which we have shown allows for the long-term engraftment of allogeneic cardiac grafts of the same haplotype as the HPCs utilized to render the mice chimeric [4]. Such work highlights the potential for ES cell-derived HPCs in transforming transplantation dynamics by reconstituting a host’s immune system with its own derivatives.

Although much understanding has been gleaned regarding the immunological properties, subpopulation characteristics, and physiological impact of ES cell-derived HPCs upon engraftment, relatively little is known about the sequence of early homing events and trafficking patterns that govern recruitment of such HPCs to various lymphoid organs. For example, it is not definitively known which organs contain the preferred microenvironments for the lodging, maturation, and establishment of ES cell-derived HPCs prior to their mobilization in peripheral blood. The bone marrow appears to be an obvious candidate due to its possession of a hematopoietic niche; however, other lymphoid organs such as the spleen, thymus, and, in mice, the extramedullary liver may contain HPC homing sites and hence be involved in the regulation of HPC trafficking [5]. Detailed analysis of niches in the bone marrow is further complicated by the osseous nature of the tissue, which limits the potential for histological analysis [6]. Unfortunately, efforts made toward real-time tracking of the dynamic behavior of ES cell-derived HPCs in vivo have been particularly inadequate because of difficulties that arise with the measurement of a small number of cells systemically dispersed across various organs [7, 8]. Therefore, a system for labeling and tracking HPCs would be beneficial in both research and clinical settings.

Mesoporous silica nanoparticles (MSNs) are often studied as vehicles for drug delivery due to their large ratios of pore volume and surface area to weight relative to other nanomaterials [9–16]. Once injected, MSNs are bioinert, nontoxic, and well tolerated at clinically relevant concentrations [17–21], unlike biodegradable polymers that can generate toxic byproducts [22–25] or nanomaterials with reactive elements such as quantum dots [26–28]. In addition, the use of MSNs in imaging applications is an area of active research. In particular, MSNs are used to label and track cells through functionalization with fluorophores [11, 29–34], ferromagnetic (iron-based) [35–37] and paramagnetic (gadolinium-based) materials [14, 21, 33, 34, 38–42] for magnetic resonance imaging (MRI), or electron-dense materials for computed tomography [43–46]. In turn, MSNs have promising utility in tracking stem cells upon their transplantation to define early trafficking patterns and to identify tissues eventually occupied by the transplanted stem cells. Although ferrous contrast agents have been used in clinical MRI applications [35–37, 47, 48], gadolinium has favorable biocompatibility and, with seven unpaired electrons in its outer shell, a higher relaxivity, particularly for T1-weighted imaging [14, 40, 42, 49–51]. Thus, it is the preferred material with which to develop innovative cell tracking schemes where high sensitivity is imperative.

Here, we describe the application of a novel MSN tool for noninvasive and longitudinal tracking of dynamic early homing patterns of transplanted ES cell-derived HPCs. The HPCs efficiently took up nanoparticles after treatment with protamine sulfate, and upon their transplantation into syngeneic mice, through non-invasive MRI, we were able to achieve high spatial and temporal resolution so as to identify the nanoparticle-labeled cells over a period of 9 days in the bone marrow, spleen, and kidneys. This modality is nontoxic and noninvasive and offers a unique leverage to evaluate homing patterns of HPCs over successive time periods in the same organism. Overall, our data indicate a striking application for MSNs for nontoxic labeling and noninvasive tracking of systemically injected cells within the same organism over a period of time.

## Methods

### Particle synthesis/characterization

Synthesis and characterization of MSNs functionalized with gadolinium oxide and either tetramethyl rhodamine isothiocyanate (Gd_2_O_3_-TRITC-MSNs) or fluorescein isothiocyanate (Gd_2_O_3_-FITC-MSNs) were obtained following previously reported syntheses [21, 33, 34, 52, 53]. Briefly, MSNs were formed by addition of tetraethyl orthosilicate (TEOS) to cetyl trimethylammonium bromide (CTAB), immediately followed by addition of Gd_2_O_3_ colloid. Next, TRITC (5.0 mg, 0.0128 mmol) was reacted with APTMS (2.2345 μl, 0.0128 mmol) in DMSO for 2 h, and Gd_2_O_3_-TRITC-MSNs were prepared by grafting 0.05 ml of the resulting product onto the previously synthesized Gd_2_O_3_-MSNs. As necessary, the particles were further functionalized with poly(ethylene glycol) (PEG) by grafting 2-(methoxy(polyethyleneoxy)propyl)trimethoxysilane (0.2 mmol) onto Gd_2_O_3_-TRITC-MSNs (100 mg) in toluene under reflux for 24 h. The resulting solution was filtered and the obtained pink solid (PEG-Gd_2_O_3_-TRITC-MSN) was washed with a copious amount of methanol and then dried under vacuum.

The materials were characterized by powder X-ray diffraction, using a Bruker D-5000 diffractometer. Surface area and porosity were analyzed using a Quantachrome NOVE e-series surface area and porosity analyzer implementing the Brunauer–Emmett–Teller (BET) equation to calculate surface area and pore volume and the Barrett–Joyner–Halenda (BJH) equation to calculate the pore size distribution. Dynamic light scattering (DLS) was carried out to obtain particle size distribution and zeta potential data, using the Malvern Zetasizer Nano ZS instrument. Samples were dispersed in 95% ethanol, and three sets of intensity-based size distributions were pooled to determine a mean diameter. Each set is comprised of a minimum of 12 measurements of approximately 10^6^ counts of light scattering. The materials were also visualized by transmission electron microscopy (TEM) by supporting samples on copper grids in a Tecnai G2 F20 microscope operating at 200 kV.

### Generation of ES cell-derived HPCs

ES cell-derived HPCs were derived according to the protocol described previously by Chan et al. [3]. Briefly, HOXB4-transduced mouse ES cells (HM1 cell line) originally derived from the 129SvJ mouse strain were grown on feeder-seeded gelatinized flasks in ES cell culture medium with 15% fetal calf serum, 1% penicillin/streptomycin cocktail (GIBCO), 0.1 mM l-glutamine, and 1000 U/ml leukemia inhibitory factor for the maintenance of pluripotency. The ES cells were then subjected to embryoid body (EB) formation. The EBs were trypsinized and dissociated into a single cell dispersion before being replated onto ultralow-attachment Petri dishes in defined medium containing StemPro34 base media plus nutrient supplement (Life Technologies/BRL) and various hematopoietic cytokines: mIL-3 (2 ng/ml), mouse stem cell factor (100 ng/ml; R&D Systems), mIL-6 (5 ng/ml), IGF-1 (40 ng/ml; Promega), Flt3-L (10 ng/ml), and dexamethasone (1 μM; Sigma-Aldrich). The cell cultures were kept in a hypoxic incubator containing 9% CO_2_.

### Cell labeling

Prior to labeling, cells were counted and the viability confirmed using trypan blue exclusion dye. In an ultralow-attachment 24-well plate, cells were suspended at 4 × 10^6^ cells/ml, with 250 μl of cell suspension added to each well. Particles (Gd_2_O_3_-TRITC-MSNs, PEG-ylated as necessary) were added at 125 μg/ml. To improve cell labeling, either polybrene or protamine sulfate was added with the particles at a final concentration of 20 μg/ml, and the cells were incubated for 15 min. An additional 250 μl of growth medium was added to reduce the cell density to 2 × 10^6^ cells/ml and the cells were incubated for a further 4 h. Because cells are not adherent to culture dishes during the labeling process, it was necessary to ensure that no free nanoparticles (NPs) were included with the cells after labeling. This was accomplished using three washes with phosphate buffered saline (PBS) and a centrifugation speed of 80 × *g* for 5 min, insufficient to pellet free NPs. Labeled cells were incubated in fresh growth medium and rewashed prior to transplantation.

### Flow cytometry

Cells were washed with PBS and fixed with 2% paraformaldehyde prior to acquisition through a BD LSR II Violet Instrument. The collected data were analyzed with FlowJo software.

### Intravenous HPC transplantation

Mice (129/SvJ) were purchased from Jackson Laboratories and housed at the vivarium located in the Veteran Affairs Medical Center, Iowa City, IA, USA. For HPC transplantations, mice were irradiated at least 24 h prior to injection with 700–800 cGy of cesium split into two doses spaced 4 h apart. On the day of transplantation, mice anesthetized with isoflourane were injected through the retro-orbital vein. This mode of injection was chosen for several reasons. First and foremost, this method of injection allows for a greater volume of the injection compared to the tail vein, avoiding NP clustering. Second, the presence of a contralateral site that was not injected allows for an internal control to be present. Finally, this method is simple and reproducible without having to subject the animal to restraints. Mice were transplanted with 7 or 14 million HPCs and 5 × 10^5^ RBC-lysed syngeneic bone marrow cells to support basic hematopoiesis until the HPCs successfully engrafted. Per established protocol [3], a small number of bone marrow cells were transplanted to sustain the animal until the HPCs engrafted and matured sufficiently to support native hematopoiesis. Mice were monitored until consciousness returned.

### Magnetic resonance imaging

Mice were scanned in the Varian® Unity/INOVA 4.7 T small animal scanner using a 25-mm gradient coil. Before and at several time points after retro-orbital injection of labeled cells, the mouse was anesthetized with isoflurane (3% induction, 1.5% maintenance) and scanned using fast spin echo (FSE) sequences (repetition time 2100 ms, echo time 15 ms with an echo train length of 8 and 5 averages per scan). Three scans were interlaced to yield images which were 256 pixels × 512 pixels with 45 slices, and a voxel size of 156 μm × 156 μm × 500 μm. Each of the three sequences had a scan time of 8 min, and an additional T2*-weighted gradient echo scan was performed for a total scan time of about 45 min per mouse. While gadolinium chelates that comprise clinical contrast agents are typically used for T1-weighted imaging, gadolinium oxide nanoparticles have moderate relaxivity in both T1 and T2-weighted images [54–56]. Thus, our scan parameters were chosen to provide an optimal combination of contrast and anatomical data.

Reconstructed images were saved as 16-bit TIF image stacks, which were opened in the free software MIPAV for analysis. Volumes of interest (VOIs) were either manually drawn or semi-automatically selected using the “levelset VOI” tool. The VOIs were drawn for each eye and each kidney, along with the liver, spleen, and long bones (the femurs and tibiae of both hind limbs), and appropriate measurements were made for each volume: number of voxels, minimum and maximum grayscale value, and average and standard deviation of grayscale values within the volume. These organs were examined due to their relevance to hematopoiesis and hence homing of the labeled cells, or drainage of the nanoparticles by themselves. Images were normalized to one another using a volume of fat adjacent to the kidneys and a small centrifuge tube of deionized water included in each scan, resulting in images consisting of floating point values largely between 0 and 1.

### Statistical analysis

After normalization, comparisons of MRI measurements between groups of mice were made using Welch’s method for the Student’s unpaired *t* test with populations of unequal variances, with an alpha level of 0.05 considered significant. Growth rates of HPCs were compared with predictive proliferative indices based on prior experience using a paired Student’s *t* test for raw cell count vs expected cell count, and an unpaired Student’s *t* test for the ratio of growth per day, with an alpha level of 0.05 considered significant.

## Results

### ES cell-derived HPCs efficiently uptake mesoporous silica nanoparticles when incubated with cationic protamine sulfate

**Fig. 1:**
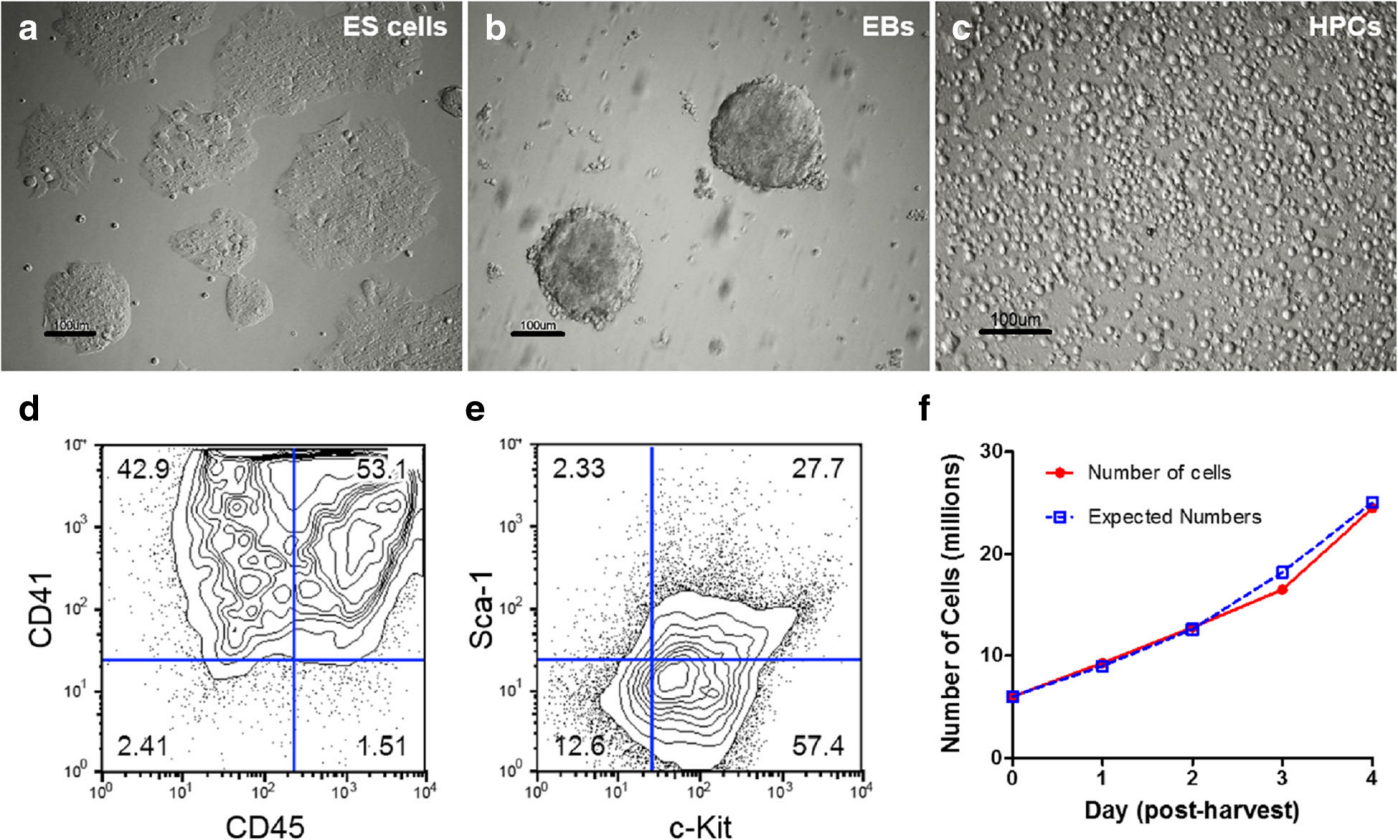
Generation and characterization of embryonic stem cell (ES) cell-derived hematopoietic progenitor cells (HPCs). Mouse ES cell colonies (**a**) coaxed to form embryoid bodies (EBs) (**b**) using methylcellulose medium, after which they are dissociated into single cells. These cells are then cultivated in a rich culture medium supplemented with growth factors to promote differentiation and propagation of HPCs (**c**).These cells strongly express hematopoietic progenitor markers CD41 and CD45 (**d**), as well as hematopoietic stem cell markers c-Kit and Sca-1 (**e**), which are thought to play roles in cell homing (**a**–**e**, *n* = 200). **f** HPCs expand at a predictable rate of ~ 1.5 times every day following harvest (observed vs predicted rate of daily turnover proliferation index, *p* = 0.141 via unpaired Student’s *t* test; *p* = 0.345 for observed vs predicted raw cell counts; *n* = 2). Scale bars = 100 μm

Our laboratories developed a series of protocols for the generation and characterization of HPCs from mouse ES cells. Following establishment, colonies are subsequently transduced with GFP-tagged HoxB4, a transcription factor that confers self-renewal capabilities to the cells and monitoring of their long-term propagation in vitro and in vivo [3]. Results are shown in (Fig. 1). Briefly, ES cell colonies (Fig. 1a) are coaxed into forming embryoid bodies (EBs) (Fig. 1b), which are dissociated and cultivated in a hematopoietic expansion medium (Fig. 1c). The cells are confirmed as HPCs by their expression of the hematopoietic progenitor cell markers CD41 and CD45 (Fig. 1d), as well as that of the hematopoietic stem cell markers c-Kit and Sca-1 (Fig. 1e). These epitopes are thought to play important roles in hematopoietic stem cell self-renewal and fate specification [57], and a role for c-Kit in homing behavior of HPCs has been described [58, 59]. These HPCs proliferate at a synchronous, predictable rate, expanding nearly 1.5-fold every day after their harvest (Fig. 1f), with no significant difference between the actual cell count and expected results based on numerous previous experiments.

**Fig. 2:**
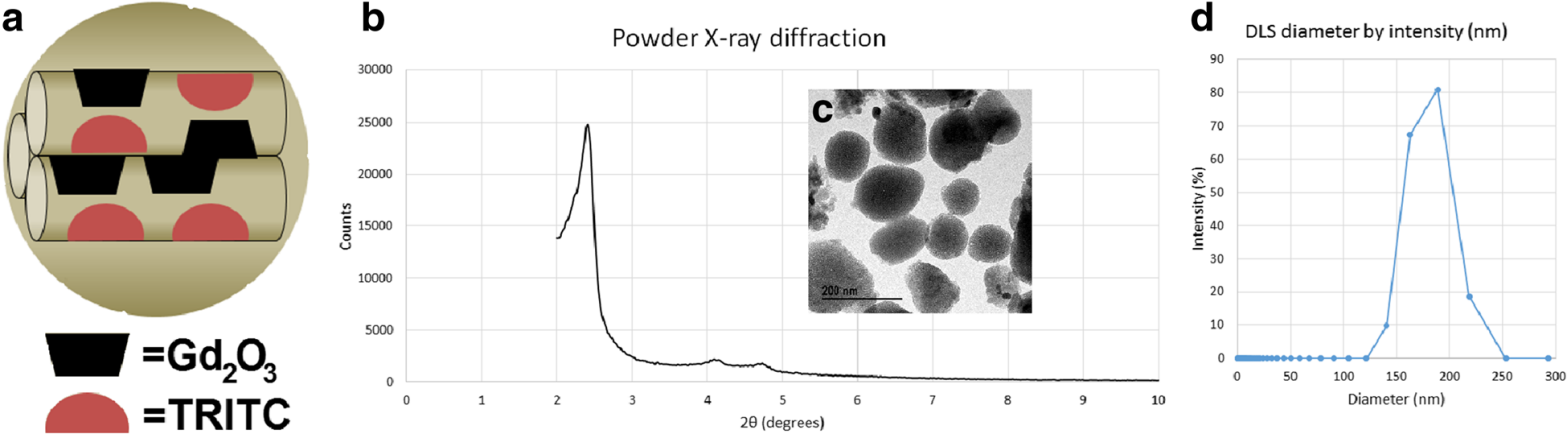
Characterization of mesoporous silica nanoparticles (MSNs). **a** Schematic showing MSNs as synthesized, with Gd_2_O_3_ incorporated into silica matrix, and surface and pore walls functionalized with tetramethyl rhodamine isothiocyanate (TRITC). As needed, particles are also functionalized with poly(ethylene glycol). **b** Powder X-ray diffraction patterns of Gd_2_O_3_-MSNs. Intense peak at 2.5 2θ characteristic of hexagonally arranged pores in MSNs, further evidenced by TEM of a particle (**c**). Visualized particles had uniform size distribution and showed no formation of aggregates. **d** Hydrodynamic size distribution of Gd_2_O_3_-TRITC-MSNs measured by dynamic light scattering. Primary peak has mean particle size of 177 nm. DLS dynamic light scattering

To label HPC with markers visible both in vitro (via fluorescence) and in vivo (via MRI contrast), we based our approach on previously used methods [21, 33, 34, 52, 53]. We modified some aspects of the protocols to generate MSNs with gadolinium oxide nanoparticles incorporated into the matrix and tetramethyl rhodamine isothiocyanate (TRITC) grafted onto the surface (Fig. 2). As needed, the particles were also grafted with poly(ethylene glycol) (PEG) to improve dispersion and cell binding properties [60]. The particles, Gd_2_O_3_-TRITC-MSNs (Fig. 2a), were characterized at each step in the synthesis. Powder XRD analysis confirmed hexagonally arranged mesopores in the diffraction pattern of the Gd_2_O_3_-TRITC-MSNs as evident by the intense d_100_, and well resolved d_110_ and d_200_ peaks characteristic for MSNs (Fig. 2b). Transmission electron micrographs of the Gd_2_O_3_-TRITC-MSNs showed this pattern as well as uniform size distributions and good dispersibility with little aggregation (Fig. 2c). Nitrogen sorption analysis of the Gd_2_O_3_-TRITC-MSNs exhibited a type-IV isotherm, typical of mesoporous materials, with a BET surface area of 710 m^2^/g. The average pore diameter for TRITC-Gd_2_O_3_-MSNs by BJH calculation is 24 Å. The fully synthesized Gd_2_O_3_-TRITC-MSNs were characterized by dynamic light scattering using ethanol as a dispersant; the mean hydrodynamic diameter of the particles was 177 nm measured on an intensity basis (Fig. 2c). The sample had a good polydispersity index (PDI) of 0.535. On this instrument, a PDI below 0.3 is considered highly monodisperse, while a PDI near 1.0 indicates a poorly dispersed or sediment-forming sample.

**Fig. 3:**
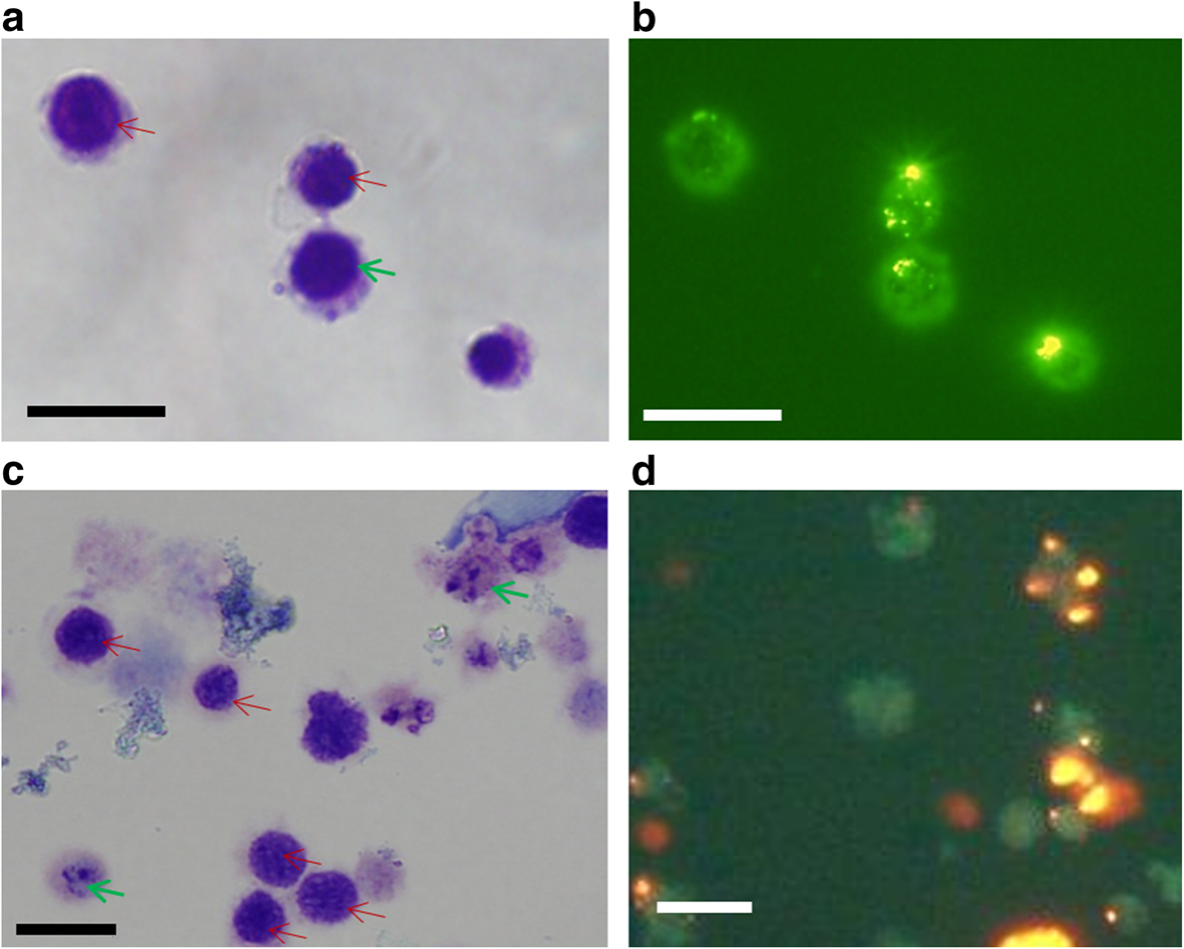
Hematopoietic progenitor cells uptake mesoporous silica nanoparticles at various stages of differentiation. **a**, **c** Wright stains: vast majority of cells are small with high ratio of nuclear to cytoplasmic volume, indicative of progenitor phenotype (red arrows), while few cells possess lower nuclear to cytoplasmic ratio and granular neutrophilic or eosinophilic cytoplasm (green arrows). **b**, **d** Fluorescent image of HPCs labeled with Gd_2_O_3_-FITC-MSNs (**b**) and Gd_2_O_3_-TRITC-MSNs (**d**), using protamine sulfate as supplement for labeling (*n* = 2). Scale bars = 10 μm

To monitor and follow the fate of HPCs both in vivo and in vitro we used specially designed multifunctional nanoparticles, detailed in Fig. 2. We adapted nanoparticles developed previously for an adherent cell population to label nonadherent HPCs. Early experiments aimed to optimize HPC labeling with MSN materials: 1.4 × 10^7^ HPCs were incubated with Gd_2_O_3_-TRITC-MSNs at 125 μg/ml and labeling evaluated using a combination of fluorescent microscopy and Wright stain. The Wright stain revealed that the vast majority of cells were mononuclear with a very high ratio of nuclear to cytoplasmic volume (Fig. 3a, c), suggestive of a progenitor cell morphology. Fluorescent micrographs confirmed the adhesion of Gd_2_O_3_-FITC-MSNs (Fig. 3b) and Gd_2_O_3_-TRITC-MSNs (Fig. 3d) to the cell surface. Flow cytometry results supported the trend observed under the microscope. While 99.8% of the cells in the negative control group were GFP^+^/TRITC^−^, the number of labeled (TRITC^+^/GFP^+^) CCE cells increased to 28% in MSNs alone, 33.7% in MSNs + polybrene, and 65% in MSNs + protamine sulfate. Quantitatively, this was an improvement over previous attempts at labeling using another cell line, HM1, in which only 5–10% of the cells were positively labeled. Other groups have reported similar cell labeling efficiencies [61]. Qualitatively, we observed a “smearing” effect on the flow cytometry plots, where many of the cells appeared to move only slightly into the positive quadrant, rather than assemble into a discrete positive population. This suggests that many of the cells that became labeled engulfed a small number of particles, which is confirmed by microscopy analysis.

**Fig. 4:**
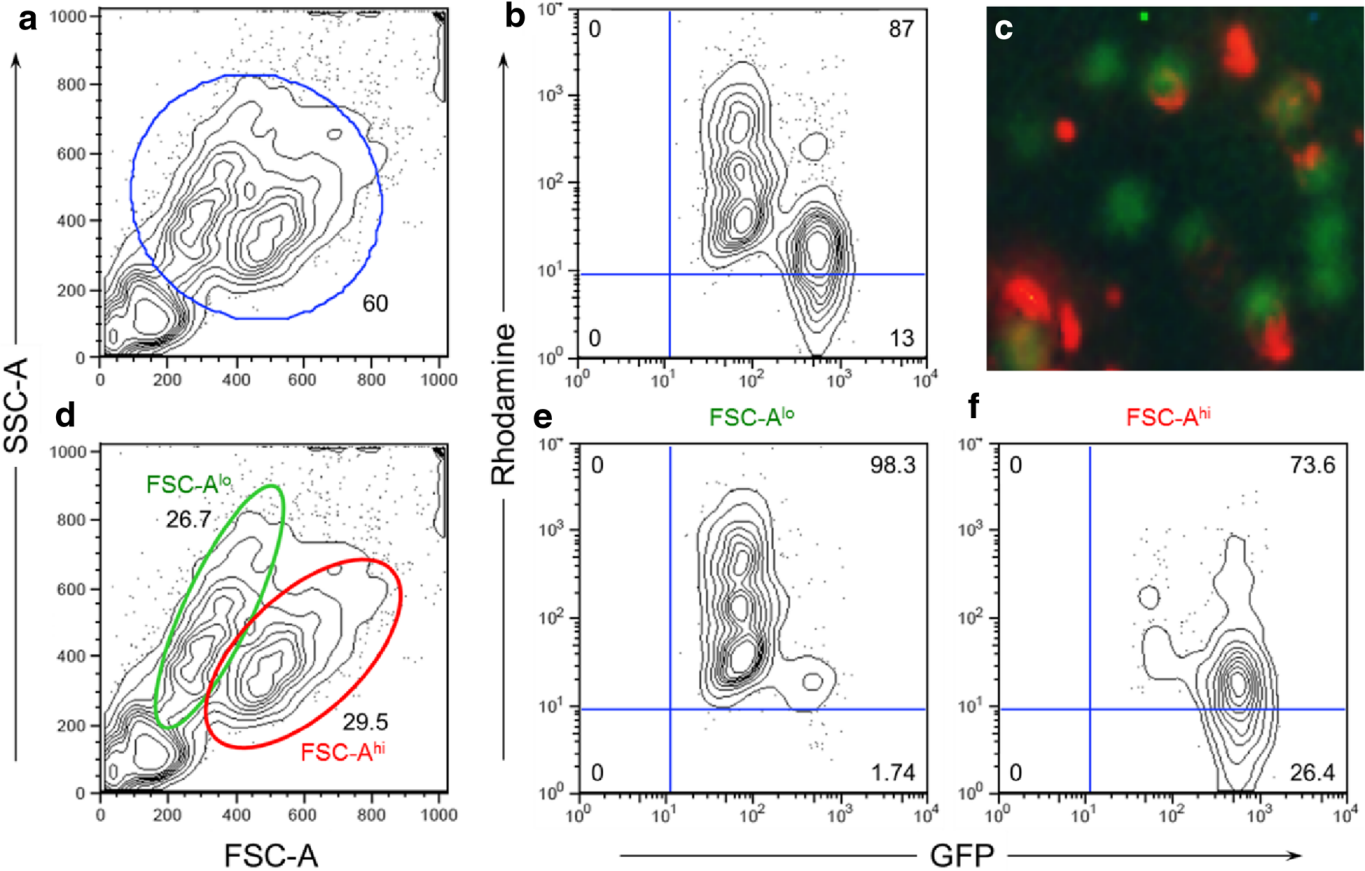
Labeling of CCE-HPCs with Gd_2_O_3_-TRITC-MSN: GFP^dim^ and GFP^hi^ HPCs show differential levels of nanoparticle uptake. **c** Composite GFP and TRITC fluorescent micrograph showing CCE-HPCs (green) labeled with MSNs containing TRITC (red). **a, b, d-f** Cytometry data for these cells, with 87% of cells showing positive signal for both TRITC and GFP (**b**). Cell population seems separated into two distinct subpopulations, explored in bottom row (**d-f**). Subpopulation marked by green oval (GFP^dim^, FSC^lo^) shown in middle panel is 98.3% TRITC^+^ with three distinct peaks of nanoparticle uptake. Subpopulation marked by red oval (GFP^hi^, FSC^hi^) is 73.6% TRITC^+^. Two subpopulations are nearly equal in size (*n* = 2). FSC forward scatter, GFP green fluorescent protein, SSC side scatter

Subsequently, we performed experiments to identify the subsets of HPCs being labeled (Fig. 4). Here, we observed 80.4% of cells to be TRITC^+^, with the overall population made up of two distinct subpopulations. The first subpopulation is lower in forward scatter, slightly higher in side scatter, and has a slightly dimmer expression of GFP relative to the second group. We have observed that GFP expression in our HPCs is downregulated as the cells become more mature (CD45^+^) and lineage committed, with evidence of preferential myeloid bias. The higher SSC and dimmer GFP expression indicates a more granulocytic morphology to the first subpopulation. This subpopulation is also 98.3% TRITC^+^ and more clearly above the threshold for positive TRITC expression, indicating superior uptake of the nanoparticles that is consistent with a more myeloid phenotype. Additionally, in this population of cells, it is evident that there are three peaks corresponding to three distinct levels of nanoparticle uptake. The second subpopulation has a higher expression of GFP but a lower level of nanoparticle uptake, with 73.6% TRITC^+^ cells and with more of the cells only slightly above the threshold. The two subpopulations are nearly equal in size, with the second group making up 52.5% of the total population.

#### Intravenous injection of labeled CCE-HPCs leads to initial MRI signal changes in eyes, kidney, and liver

Three female 129 SvJ mice (age 4 weeks) were injected with 7.2 × 10^6^ labeled CCE-HPCs, plus 5 × 10^5^ syngeneic bone marrow cells, in the left retro-orbital plexus. Mice were scanned before the injection and 3 h later, and then on the following day and twice more on days 6 and 9. After reconstruction/normalization, the value of the right eye was divided by that of the left at each time point, and the difference between each point and the “before” time point was plotted (see Additional file 1). Relative to the “before” scan, hypointensity was observed in the left eye relative to the right eye in the 3-h scan. Additionally, the total volume of interest measured in this location increased from an average of 5.4 μl at other time points to 8.5 μl. In subsequent scans, the normalized values of the two eyes were virtually identical. The average difference at 3 h was compared to zero, although the difference did not achieve statistical significance (*p* < 0.2).

Like the eyes, darkening of the renal vasculature was observed at 3 h following injection. Compared with the eyes, the hypointensity seemed to diminish more slowly as the experiment continued (see Additional file 2), taking a full 9 days to decrease to the same level as what was observed before the injection. The hypointensity was observed equally in both kidneys, and the volume of interest increased from 10.9 μl before the injection to 17.6 μl 3 h after the injection, a 61% increase in volume. This suggests that 61% more of the renal vasculature could be detected because of the improvement in contrast after the injection. When data from three mice were pooled, a significant difference could not be established between any of the points and the “before” scan. Comparing the mean at *t* = 1 day with that at *t* = 9 days did result in a significant difference (*p* < 0.01).

A similar pattern of steadily decreasing signal after the 3-h time point could be observed in the liver (see Additional file 3). The magnitude of the change (peaking at about 5% on a normalized scale from 0 to 1) was similar as well. Unlike the kidneys, where the signal appeared to be confined to the renal vasculature, the signal change in the liver was more global, with a decrease in the average grayscale value of the entire organ. After subtracting each time point from the “before” scan and averaging the differences, significant changes were observed on day 1 (*p* < 0.1) and day 6 (*p* < 0.01).

### Evidence in mouse model of signal change as labeled cells accumulate in spleen and bone marrow

**Fig. 5:**
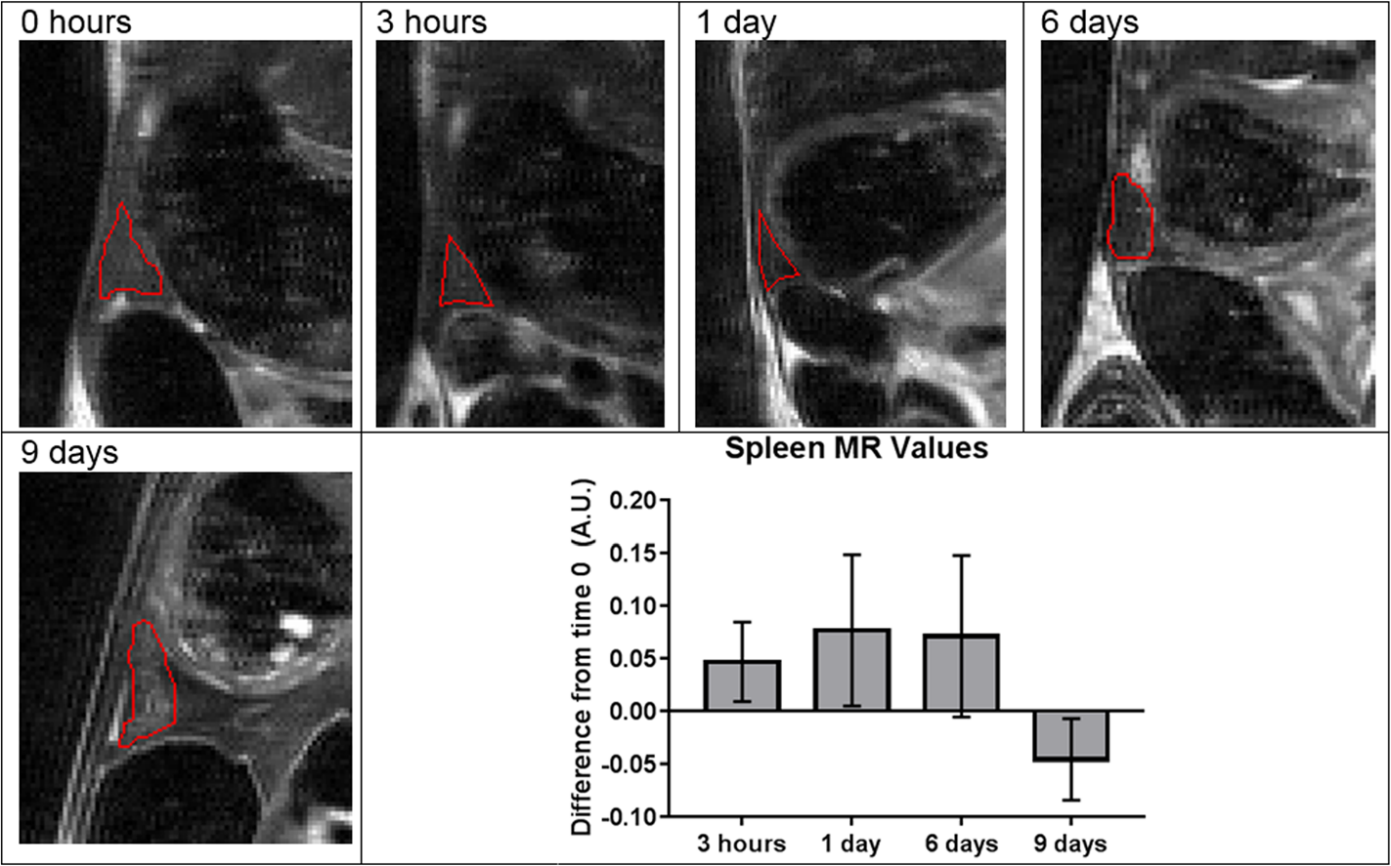
Magnetic resonance intensity changes in spleen following injection of labeled CCE-HPCs. MRI of spleen (outlined in red, with stomach superior and large intestine inferior) in mouse before and at various time points following injection with 7 × 10^6^ labeled CCE-HPCs. Spleen steadily darkens following injection, and darkening diminishes as experiment continues. Plot shows average normalized MR value at each time point, with error bars indicating standard deviation. Although a trend of signal hypointensity followed by return to original value was observed, statistically significant differences were not achieved (*n* = 3). A.U. arbitrary units, MR magnetic resonance

Four hours and 1 day following intravenous injection of labeled CCE-HPCs in the retro-orbital cavities of mice, we observed MRI signal changes in several tissues, including the eyes, kidneys, and liver (see Additional files 1, 2 and 3). Qualitatively, hypointensity is also observed in the spleen and bone marrow, but with a delayed onset, suggesting that the labeled cells circulated for several days until they accumulated with sufficient density to be seen. In the spleen, hypointensity increases over time, suggesting accumulation of MSN material between 1 and 6 days; by 9 days it returns to the grayscale level observed prior to injection (Fig. 5). Quantitative analysis was also performed; when the two mice were averaged, changes appeared to be within the standard deviation and no pairwise comparisons showed a significant difference. Attempts were made to measure volumetric changes in the spleen as well, but no trends were observed, as it was difficult to reliably determine the border zone of the spleen with the changing shape and intensity of the surrounding stomach and large intestine.

**Fig. 6:**
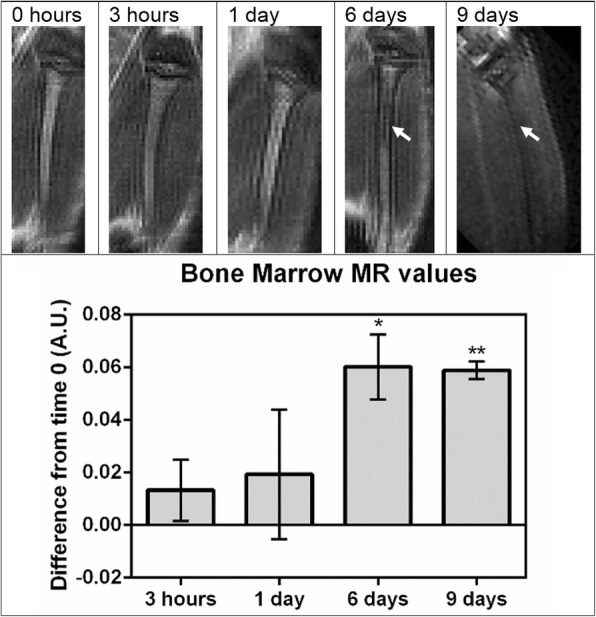
Magnetic resonance intensity changes in bone marrow following injection of labeled CCE-HPCs. MRI of right tibia in mouse before and at various time points following injection with 7 × 10^6^ labeled CCE-HPCs. Slices selected to display equivalent regions in right tibia, where bone marrow is observed to darken 6–9 days following injection (arrows). Both tibias and femurs of three mice used in measurements shown. Next, difference between each time point and “before” scan calculated by subtraction. Plot shows average of this difference from “before” scan, with error bars indicating standard deviation. Comparing in this manner, significant differences observed for day 6 (*p < 0.1) and day 9 (**p < 0.05) (*n* = 3). A.U. arbitrary units, MR magnetic resonance

**Fig. 7:**
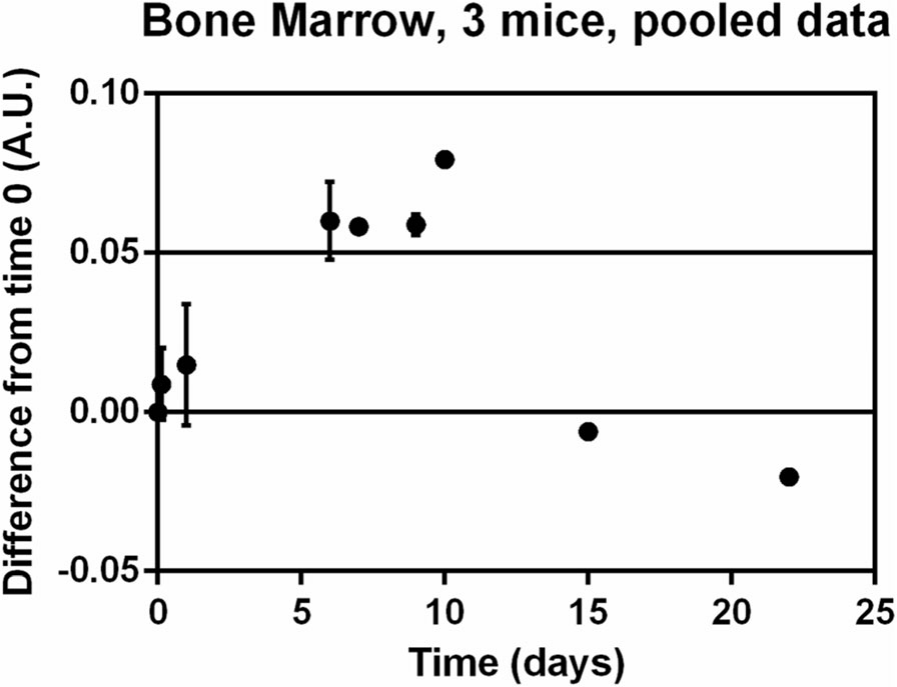
MRI of bone marrow indicates homing of hematopoietic progenitors, followed by signal loss. Change in normalized grayscale value plotted as difference from time *t* = 0 for three mice. Difference between data points and time *t* = 0 appeared to increase to maximum between 6 and 10 days after injection, then return to values seen at start of experiment. Error bars indicate standard deviation for time points at which more than one mouse was scanned (*n* = 3). A.U. arbitrary units

In the bone marrow, a similar signal change peaking at about 5% was observed, but the onset of the signal change was delayed (Fig. 6). On days 6 and 9 the signal changes were apparent to the naked eye. The right tibia is shown in the figure, but both tibias and femurs were used in quantitative analysis, and a similar pattern of darkening was observed in the marrow of all bones. The results were consistent between mice as well; with three mice, significant differences were observed between the initial scan and the 9-day scan (*p* < 0.1), and between the 1-day and 6-day scans (*p* < 0.05). Additionally, when the difference between each time point and the initial scan was calculated for each animal and then averaged, the 9-day scan was significantly different from 0 (*p* < 0.05). Lastly, the three datasets were pooled so that a trendline could be depicted (Fig. 7) and regression analysis was performed. The difference between the data points and time *t* = 0 appeared to increase to a maximum between about 6 and 9 days after the injection, and then returned to the values seen at the start of the experiment.

We noninvasively monitored HPC trafficking for 9 days in the same mice with a scanning regimen detailed in Figs. 6 and 7 (3 h–9 days). Ultimately, mice were sacrificed 20 days following labeled HPC injection. At this point, blood samples were drawn for evaluation of chimerism via GFP expression. These samples indicated that chimeras did not reach detectable levels. This may suggest a low long-term engraftment below detection had taken place in the bone marrow.

## Discussion

Here, we demonstrated a noninvasive modality to detect the initial homing and reconstitution patterns of stem cell-derived HPCs within various organ compartments relevant to hematopoiesis, namely the spleen, bone marrow, and liver. In the bone marrow, we detected early and sustained signals changes consistent with the early signs of engraftment onset. These data could have alternatively been obtained with flow cytometry analysis of bone marrow cells, but that would require sacrifice of the animal. Postmortem experimentation would have hindered further important evaluation of the whole animal. Instead, our technology can serve as an excellent complement to other methods in tracking transplanted cells, especially within transplanted animals that need to be kept viable for future experimental study. It is unique in that we are able to carry out a “virtual biopsy” of many internal organs for preliminary analysis of early engraftment patterns of the HPCs without sacrificing the animal. This allows computational serial imaging examination of the same animal for various functional studies that could follow engraftment with stem cell-derived HPCs. Prior work in our laboratory has shown how flow cytometry can demonstrate an identifiable population of stem cell-derived HPCs to engraft in bone marrow within 24 h post transplantation [62]. In contrast, this MRI modality of detecting engraftment seems to require more time to fully provide evidence of bone marrow engraftment. While flow cytometry allows for identification of the small population that does reach the bone marrow within 24 h, MRI mandates a longer duration of homing, engraftment, and expansion before the cells are able to be identified. That being said, the cons of MRI in this case are far outweighed by the noninvasive modality of imaging in confirming engraftment on a superficial level, when specific cell markers are not needed.

We pursued a unique translational vision to combine biotechnology and regenerative medicine not only to generate and transplant ESC-HPCs, but to allow for noninvasive tracking of the cells to interrogate their early homing patterns in a way that could not have been achieved via any other modality. To achieve this goal, we developed novel nanoparticles with components for imaging via fluorescent and magnetic resonance imaging, improving the detection of labeled versus nonlabeled cells. Taking advantage of this, we observed through manual counting methods that nearly 50% of the cells treated with only MSNs are associated with at least one pixel containing particles detectable by the naked eye. The 2× protamine sulfate treatment increases the percentage to about 65%; these findings are consistent with those of Janic et al. [61], who used protamine sulfate to enhance uptake of iron oxide particles in purified CD34^+^ hematopoietic stem cell populations. The Janic group observed a binding efficiency of 37.6 ± 15.8% using their methods; the fact that we included PEG-ylated MSNs and induced stem cells may account for our higher binding efficiency, which is only slightly higher than their margin of error.

It is unclear why the HM1-HPC line showed less propensity to being labeled by MSNs compared to the CCE-HPC line. HM1 cell viability immediately after labeling sometimes dropped below 80%, and the cells did not recover well. At the time of injection, the lowest viability of the four groups tested using CCE-HPCs was 89% for MSNs + protamine sulfate, more than sufficiently viable for experiments to be carried out. Because of their higher propensity to acquire nanoparticle labeling, we carried out subsequent experiments on the CCE-HPCs supplemented with protamine sulfate. These differences between cell lines may be attributed to variations in cell morphology, as hematopoietic cells are diverse and differentiations of different ES cell lines may yield HPCs with various lineage biases with different propensities for particle uptake.

In addition to verifying the nontoxicity of the MSNs in vitro, we previously demonstrated the biocompatibility of our MSN materials in adult and fetal mouse models [21]. In these studies, we administered MSNs intravenously in gravid mice and assessed many potential effects of exposure: histopathological findings of maternal liver and kidneys, production of inflammatory cytokines/chemokines, reactive oxygen species, and fetal developmental defects. Compared with control litters, the embryonic and placental weights and litter sizes were equivalent in MSN-exposed mice. An assay for 23 cytokines/chemokines and reactive oxygen species showed only granulocyte-colony stimulating factor was produced at a significantly higher value in exposed versus control mice. Mild microvesicular hepatocellular vacuolation was observed in the livers of both exposed and control mice, and no notable pathology was observed in the kidneys. We concluded that dams and pups were not affected by nanoparticle exposure. Litter sizes and embryo weights were equivalent between groups, and mice carried to term were free from developmental defects [21]. Based on these earlier findings, we were confident we could carry out this study without adverse effects in vivo.

When comparing the microscopy images with the flow cytometry results, it appears that a large majority of the cells only increased in TRITC expression enough to just go over the threshold. The flow cytometer has much more sensitive optics and can pick up smaller fluorescence signals than the microscope, under which it appears that much fewer of the cells are positively labeled. In other words, most of the labeled cells are labeled at a relatively low level, perhaps even with just a few individual particles. To be able to see the cells after a systemic injection, it is imperative that they have places to home to and accumulate in sufficiently large quantities so as to be seen using MRI. In our experiments, we expected to inject up to 20 million labeled cells; however, we were limited by the retro-orbital injection to a volume of about 100 μl, and 15 million cells at this density clogged the syringe needle being used. In future experiments, a larger volume of cells may be injected via tail vein injection at the cost of losing our internal control of an injection and contralateral retro-orbital space.

In a subsequent trial, we observed two distinct populations of TRITC^+^/GFP^+^ cells: TRITC^+^/GFP^high^ and TRITC^+^/GFP^dim^. The cells that are GFP^dim^ appear to be expressing higher levels of TRITC, which would indicate that they are labeled with more particles relative to the GFP^high^ cells. It is believed that the GFP^dim^ cells may have a more mature hematopoietic character (CD41^+^CD45^+^, not just CD41^+^) than the GFP^high^ cells. If so, it is possible that the CD45^+^GFP^dim^ cells have more granulocytic features that enable them to engulf the particles with higher efficiency.

In analyzing the MRI scans, we first compared the retro-orbital space of the left eye (the side that was injected with labeled cells) with the contralateral side. Thus, the calculation applied to each voxel *I*_(*x*, *y*, *z*, τ)_ comprising the left retro-orbit for each time point (τ) is ${I}_{\left(x,y,z,\tau \right)}=\left(\frac{Raw\  MR\ {Value}_{\left(x,y,z,\tau \right)}}{\overline{I_{right\  eye,\tau }}}\right)-\left(\frac{Raw\  MR\ {Value}_{\left(x,y,z,0\right)}}{\overline{I_{right\  eye,0}}}\right)$. The injection site was relatively hyperintense 3 h following the injection, followed by hypointensity from 24 h to 8 days. When each image was normalized onto a scale from 0 to 1, where 0 represents the darkest part of the scan and 1 represents the brightest (fat deposits adjacent to the kidneys), the difference between the two sides is 1.4% before the injection (when we expect the values to be identical). At 3 h, the injection side is 3.1% brighter than the contralateral side, which may be attributable to some bleeding of the injection site, as pathological blood or other fluid tends to show up brightly in T2-weighted images. At 24 h following the injection, the left eye is 4.9% darker than the contralateral side. The left eye was 3.9% darker at 6 days and 7.2% darker at 8 days.

Our primary aim in this article was to demonstrate the use of these particles to show bone marrow homing of labeled stem cells following retro-orbital injection. In the first 24 h of scanning, we did observe an MRI signal attenuation (darkening) in both the kidneys and liver. At these locations, we expect to see accumulation of loose/free particles more so than intact cells, and while most of the particles were bound to cells, removal of all of the unbound particles is challenging. We describe in detail the tracking of nanoparticles in adult and fetal mice [21]. In addition, Choi et al. [63] investigated glomerular filtration rates of quantum dots, showing that larger particles (greater than 10 nm) are filtered much more slowly than small particles. Our 177-nm particles would likely accumulate in the Bowman’s capsule, located in the kidney cortex. The MRI data support this notion quantitatively, although the difference between the “before” scan and the 3-h and 24-h scans was not found to be statistically significant (*n* = 3). In future studies, we are warranted to expand the scope of the experiment and examine this phenomenon further.

In the bone marrow, the normalized MRI data suggested an accumulation of contrast around day 7 followed by a tapering phase thereafter. The homing of the HPCs to the bone marrow is a welcomed confirmation of the anticipated outcome based on their expression of receptors that engage with ligands expressed there. In other words, the HPCs are shown to express c-Kit (CD117), the receptor for stem cell factor (SCF), which is known to be expressed in and functions to support hematopoiesis and guide HPCs to appropriate niches in the bone marrow and liver [59]. Importantly, labeling of HPCs with the nanoparticles does not appear to have had an impact on their expected homing patterns. This may suggest that our nanoparticle configuration and composition is appropriate for this application of long-term accurate tracking of homeostatic trafficking of systemically transplanted cells. It is also important to note that based on our in-vitro labeling experiments we do not expect particles to be excreted by viable cells. Consequently, any free MSN in the circulation is assumed to be processed by the filter organs, such as the liver and kidney [21]. In addition we believe that, while we have MRI evidence to show homing of labeled cells to the bone marrow and early reconstitution patterns, long-term chimerism was not established in these mice due to their lack of exposure to natural killer (NK) cell-depleting antibody treatment. We have shown that the self-renewing c-Kit^+^Lin^−^ HPC subpopulation, a critical step for establishing long-term HPC residence in the bone marrow, is susceptible to deletion by radioresistant NK cells [64], as recently confirmed by our group [62]. Beyond the scope of this study, we considered that experiments using preconditioned mice with NK cell-depleting antibody treatments could perturb homing behavior and influence early engraftment changes as evidenced by MRI. The research presented may serve as an exciting harbinger of follow-up studies in which long-term reconstitution changes can be investigated noninvasively (in the same animal) using MRI for tracking of the labeled cells. Keeping future work in mind, our purpose in this study was to investigate early homing patterns, in which MRI has the most utility, rather than once hematopoiesis and cell reconstitution into the bone marrow have stabilized, at which point signal changes are unlikely to be detected.

## Conclusions

In this article, we report on the new option of a nontoxic, noninvasive modality for longitudinal tracking and real-time imaging of ES cell-derived HPCs during the early phases of engraftment. Our data show that the cells predominantly occupy the bone marrow over the course of the 9-day period studied in this report, but also localize in the spleen and liver in the days immediately following transplantation. The insight gained from these studies suggests that mobilization of the HPCs in preliminary phases of reconstitution is followed by lodging of the HPCs within the preferred niches of the bone marrow, where the cells are meant to populate long term for hematopoietic maintenance. Most significantly, we have validated a novel technological application for MSNs as noninvasively detectable, long-term tracers of single blood-borne cells injected systemically in vivo. These results implicate the potential utility for MSNs in tracking single cancer cell metastases and subsequent seeding into distant organs, and may help in understanding the behavior and tendencies of such patterns of dissemination and colonization of certain cancer cells.

## Additional files

Additional file 1:MRI signal changes observed near the eye following retro-orbital injection of CCE-HPCs labeled with Gd_2_O_3_-TRITC-MSNs. MRI of retro-orbital injection site in a mouse before and at various time points following injection with 7 × 10^6^ labeled CCE-HPCs. Injection in left eye (green) and right eye (red) used as control. Images normalized using olfactory sinus and an Eppendorf tube of water as low and high points, respectively, and difference between each time point and “before” scan calculated by subtraction. Thus, calculation applied to each voxel *I*_(*x*, *y*, *z*, τ)_ for each time point (τ) is ${I}_{\left(x,y,z,\tau \right)}=\left(\frac{Raw\  MR\ {Value}_{\left(x,y,z,\tau \right)}}{\overline{I_{right\  eye,\tau }}}\right)-\left(\frac{Raw\  MR\ {Value}_{\left(x,y,z,0\right)}}{\overline{I_{right\  eye,0}}}\right)$. Error bars indicate standard deviation. At 3 h, *p* < 0.2 when compared with 0 (*n* = 3). (TIF 1254 kb)

Additional file 2: Contrast enhancement in renal vasculature following injection of labeled CCE-HPCs. MRI of right kidney in mouse before and at various time points following injection with 7 × 10^6^ labeled CCE-HPCs. Slices selected to display equivalent regions in each kidney. Renal vasculature (arrows) darkens following injection, and darkening diminishes as experiment continues. Plot shows average normalized MR value at each time point for three mice, with error bars indicating standard deviation. *Comparing mean at 1 day and 9 days resulted in a significant difference (*p* < 0.01). (TIF 2549 kb)

Additional file 3: Magnetic resonance intensity changes in the liver following injection of labeled CCE-HPCs. MRI of liver in mouse before and at various time points following injection with 7 × 10^6^ labeled CCE-HPCs. Liver steadily darkens following injection, and darkening diminishes as experiment continues. Plot shows average of this difference from “before” scan, with error bars indicating standard deviation (*n* = 3). Significant changes observed 1 day (*p < 0.1) and 6 days (**p < 0.01) after injection. (TIF 1714 kb)

## Abbreviations

BET: Brunauer–Emmett–Teller
BJH: Barrett–Joyner–Halenda
CTAB: Cetyl trimethylammonium bromide
DLS: Dynamic light scattering
EB: Embryoid body
ES: Embryonic stem
FITC: Fluorescein isothiocyanate
FSE: Fast spin echo
GvHD: Graft versus host disease
HPC: Hematopoietic progenitor cell
HSC: Hematopoietic stem cell
MHC: Major histocompatibility complex
MRI: Magnetic resonance imaging
MSN: Mesoporous silica nanoparticle
NK: Natural killer
NP: Nanoparticle
PBS: Phosphate buffered saline
PEG: Poly(ethylene glycol)
SCF: Stem cell factor
TEM: Transmission electron microscopy
TEOS: Tetraethyl orthosilicate
TRITC: Tetramethylrhodamine isothiocyanate
VOI: Volume of interest
