# Supplementary figures and images for: Tracking embryonic hematopoietic stem cells to the bone marrow: nanoparticle options to evaluate transplantation efficiency
Source: Stem Cell Res Ther. 2018 Jul 27;9:204. doi: 10.1186/s13287-018-0944-8 (PMC6062968; doi:10.1186/s13287-018-0944-8)

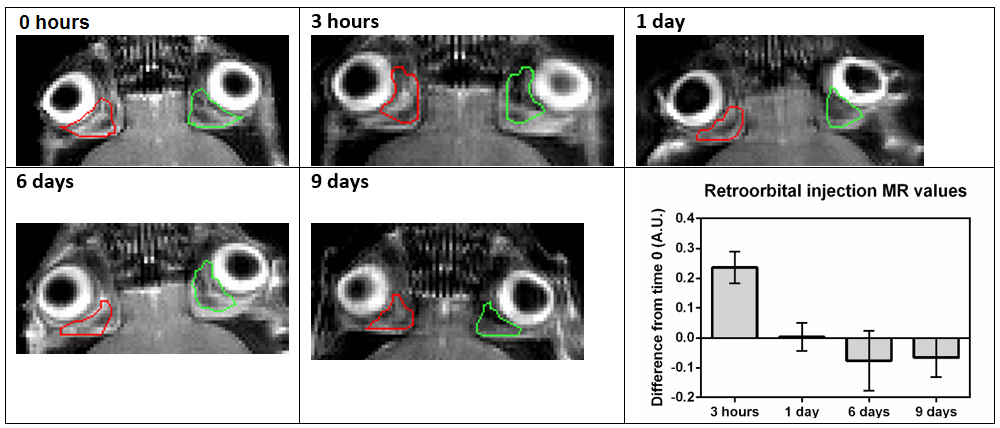

Supplement: Supplementary file 1 — MRI signal changes observed near the eye following retro-orbital injection of CCE-HPCs labeled with Gd2O3-TRITC-MSNs. MRI of retro-orbital injection site in a mouse before and at various time points following injection with 7 × 10^6^ labeled CCE-HPCs. Injection in left eye (green) and right eye (red) used as control. Images normalized using olfactory sinus and an Eppendorf tube of water as low and high points, respectively, and difference between each time point and “before” scan calculated by subtraction. Thus, calculation applied to each voxel I(x, y, z, τ) for each time point (τ) is ${I}_{\left(x,y,z,\tau \right)}=\left(\frac{Raw\ MR\ {Value}_{\left(x,y,z,\tau \right)}}{\overline{I_{right\ eye,\tau }}}\right)-\left(\frac{Raw\ MR\ {Value}_{\left(x,y,z,0\right)}}{\overline{I_{right\ eye,0}}}\right)$Ixyzτ=RawMRValuexyzτIrighteye,τ¯−RawMRValuexyz0Irighteye,0¯. Error bars indicate standard deviation. At 3 h, p < 0.2 when compared with 0 (n = 3). (TIF 1254 kb) [file 13287_2018_944_MOESM1_ESM.tif]

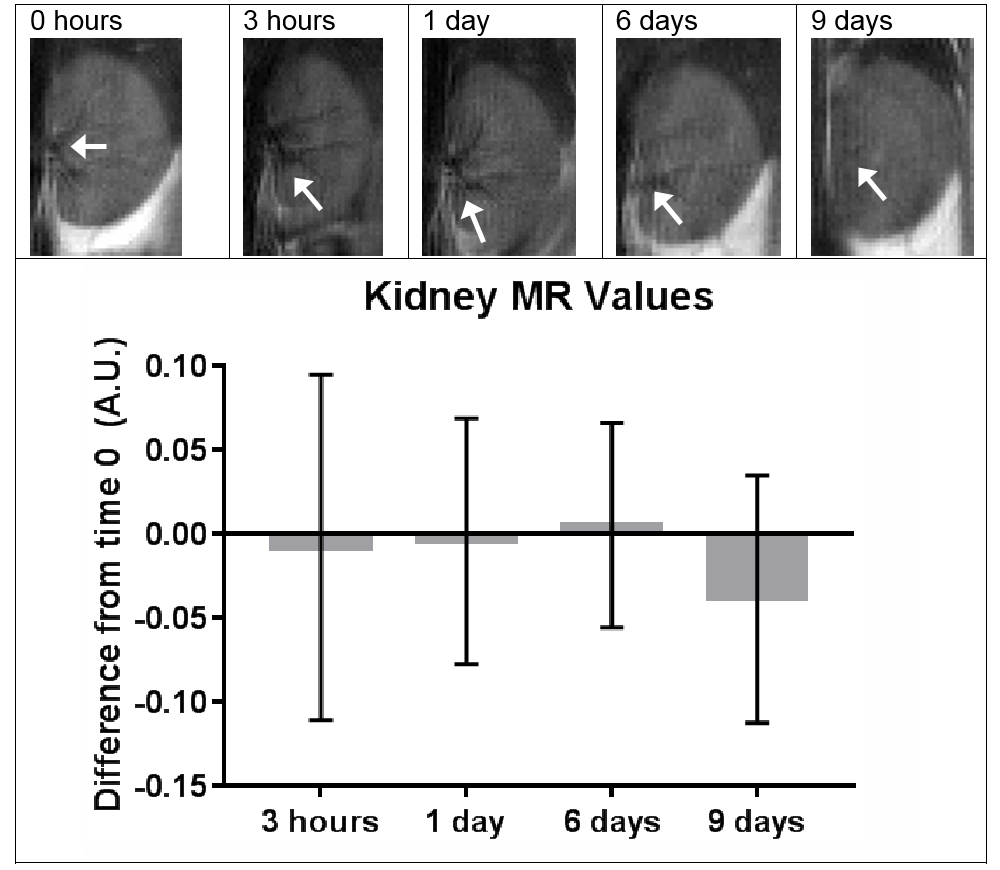

Supplement: Supplementary file 2 — Contrast enhancement in renal vasculature following injection of labeled CCE-HPCs. MRI of right kidney in mouse before and at various time points following injection with 7 × 10^6^ labeled CCE-HPCs. Slices selected to display equivalent regions in each kidney. Renal vasculature (arrows) darkens following injection, and darkening diminishes as experiment continues. Plot shows average normalized MR value at each time point for three mice, with error bars indicating standard deviation. *Comparing mean at 1 day and 9 days resulted in a significant difference (p < 0.01). (TIF 2549 kb) [file 13287_2018_944_MOESM2_ESM.tif]

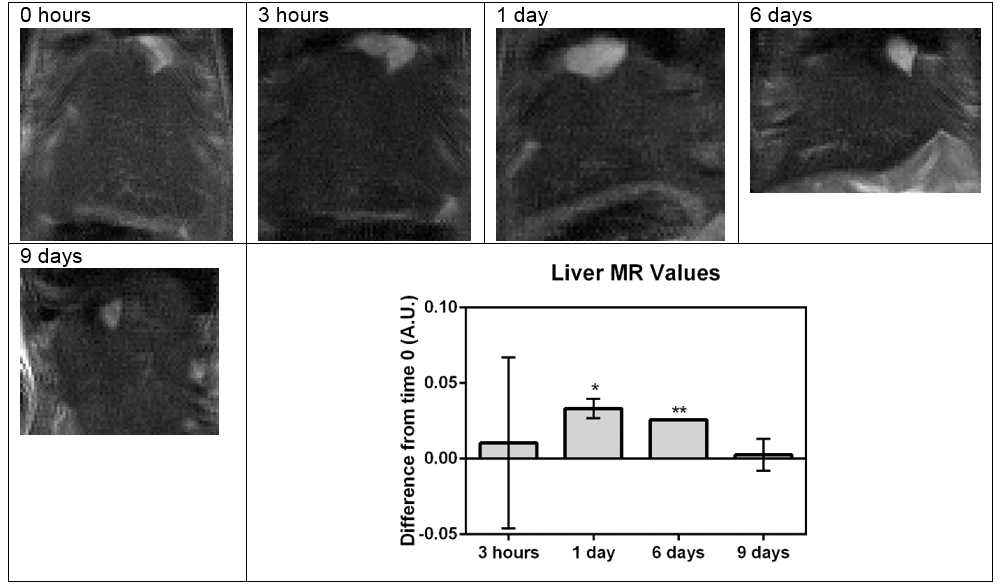

Supplement: Supplementary file 3 — Magnetic resonance intensity changes in the liver following injection of labeled CCE-HPCs. MRI of liver in mouse before and at various time points following injection with 7 × 10^6^ labeled CCE-HPCs. Liver steadily darkens following injection, and darkening diminishes as experiment continues. Plot shows average of this difference from “before” scan, with error bars indicating standard deviation (n = 3). Significant changes observed 1 day (*p < 0.1) and 6 days (**p < 0.01) after injection. (TIF 1714 kb) [file 13287_2018_944_MOESM3_ESM.tif]
